# Estimates of adherence and error analysis of physical activity data collected via accelerometry in a large study of free-living adults

**DOI:** 10.1186/1471-2288-8-38

**Published:** 2008-06-09

**Authors:** David R Paul, Matthew Kramer, Kim S Stote, Karen E Spears, Alanna J Moshfegh, David J Baer, William V Rumpler

**Affiliations:** 1 Diet and Human Performance Laboratory, Beltsville Human Nutrition Research Center, Agricultural Research Service, United States Department of Agriculture, 307B Center Rd., Beltsville, MD 20705, USA; 2 Department of Health, Physical Education, Recreation, and Dance, University of Idaho, P.O. Box 442401, Moscow, ID 83844, USA; 3 Biometrical Consulting Service, Agricultural Research Service, United States Department of Agriculture, 10300 Baltimore Ave., Building 005, Beltsville, MD 20705, USA; 4 Food Surveys Research Group, Beltsville Human Nutrition Research Center, Agricultural Research Service, United States Department of Agriculture, 10300 Baltimore Ave., Building 005, Beltsville, MD 20705, USA

## Abstract

**Background:**

Activity monitors (AM) are small, electronic devices used to quantify the amount and intensity of physical activity (PA). Unfortunately, it has been demonstrated that data loss that occurs when AMs are not worn by subjects (removals during sleeping and waking hours) tend to result in biased estimates of PA and total energy expenditure (TEE). No study has reported the degree of data loss in a large study of adults, and/or the degree to which the estimates of PA and TEE are affected. Also, no study in adults has proposed a methodology to minimize the effects of AM removals.

**Methods:**

Adherence estimates were generated from a pool of 524 women and men that wore AMs for 13 – 15 consecutive days. To simulate the effect of data loss due to AM removal, a reference dataset was first compiled from a subset consisting of 35 highly adherent subjects (24 HR; minimum of 20 hrs/day for seven consecutive days). AM removals were then simulated during sleep and between one and ten waking hours using this 24 HR dataset. Differences in the mean values for PA and TEE between the 24 HR reference dataset and the different simulations were compared using paired *t*-tests and/or coefficients of variation.

**Results:**

The estimated average adherence of the pool of 524 subjects was 15.8 ± 3.4 hrs/day for approximately 11.7 ± 2.0 days. Simulated data loss due to AM removals during sleeping hours in the 24 HR database (n = 35), resulted in biased estimates of PA (p < 0.05), but not TEE. Losing as little as one hour of data from the 24 HR dataset during waking hours results in significant biases (p < 0.0001) and variability (coefficients of variation between 7 and 21%) in the estimates of PA. Inserting a constant value for sleep and imputing estimates for missing data during waking hours significantly improved the estimates of PA.

**Conclusion:**

Although estimated adherence was good, measurements of PA can be improved by relatively simple imputation of missing AM data.

## Background

The benefits of physical activity (PA) on the reduction of risk of developing many chronic diseases [1-3] have lead to recommendations that the public should increase moderate intensity PA to a minimum of 30 – 60 min/day [2,4,5]. Despite the importance placed on investigating the effects of PA, scientists continue to struggle with the complexities associated with quantifying it [6], particularly using one of the many traditional measurement techniques (such as questionnaires, PA records and recall diaries) [6-10]. As an alternative to traditional survey techniques, activity monitors (AM) have been increasingly utilized by investigators [11] in PA studies of children and adults [12-14].

Activity monitors are small, electronic devices worn by subjects that can continuously measure the bodily movements of subjects for days or weeks at a time. One disadvantage of these devices is that subjects may remove the AMs periodically (during sleep, bathing, and non-compliance), which has been shown to impact the prediction of both total accumulated and average PA [15]. Catellier et al. [15] were among the first authors to not only recognize the pitfalls of missing AM data, but to also describe a procedure that reduces biases in the estimates of PA by imputing missing data. Their study was based on the results of a large group of adolescent girls (n = 436), whose estimated adherence to wearing AM's was approximately 12 ± 4 hrs/day during the course of seven days [15].

At present, little is known about what adherence estimates can be expected from a large group of adults, the effect missing data may have on the prediction of PA, or the effectiveness of imputation on the estimation of both PA and total energy expenditure (TEE). Generally, investigators do not report AM adherence, how missing data were treated, and/or how much data (hrs/day) were considered acceptable to included in the analysis [13,14,16-24]. In this study, we were interested in: (1) estimating the adherence of AM wear in a large study of adults, (2) determining the magnitude of biases and variability resulting from missing data in estimates of PA and TEE, and (3) exploring procedures for reducing the bias if missing data appear to be problematic. Our *a priori *hypotheses were that adherence estimates would be rather strong (approximately 16 hrs/day), but the predictions of PA would be negatively influenced by missing data.

## Methods

### Subjects

The subjects in this study were 524 women and men from the Baltimore, MD/Washington, DC area (Table 1). The data were collected continuously from July 2002 to August 2003. The subjects received an honorarium for completing the study.

**Table 1:** Characteristics of the subjects (n = 524).

	Male (n = 262)	Female (n = 262)
	%	%
Age (y)		
30–39	21	23
40–49	28	26
50–59	26	29
60–70	24	21
Race/Ethnicity		
Non-Hispanic White	83	71
Non-Hispanic Black	7	19
Hispanic	3	3
Other	6	7
Education		
High school diploma or less	4	10
Some college to Bachelor degree	49	59
Graduate degree	47	31
BMI (kg/m^2^)		
< 25.0 (Normal)	36	48
25.0–29.9 (Overweight)	44	30
≥ 30.0(Obese)	21	21

The study protocol was approved by the Johns Hopkins University Bloomberg School of Public Health Committee on Human Research. Prior to participation, subjects provided written informed consent and received a medical evaluation by a physician that included measurement of blood pressure and analysis of fasting blood and urine samples to screen for presence of metabolic disease (such as diabetes). Body composition (lean and fat mass) was measured by DEXA (QDR 4500), with lean body mass values adjusted according to Schoeller et al. [25].

### Activity monitoring

The AM (Actigraph 7164; Manufacturing Technology, Inc. Fort Walton Beach, FL) was worn on a snuggly-fitting waist belt (according to the manufacturer's instructions), with the manufacturer's "notch" facing upwards. The AM was set to store the data in 1-min intervals of time (epochs). The technical details of this brand of AM have been described elsewhere [26]. Subjects were asked to wear the AM on the right hip, unless they reported being unable to do so. Regardless of the AM placement on the hip (left or right), each individual consistently wore it on the same side and location. The subjects were asked to wear the AM continuously for 13–15 days, depending on study scheduling. In addition to wearing the AM, subjects were asked to maintain an activity log (modified from [27]) that detailed when they went to bed, woke up, removed it (and why), and detailed any structured exercise they may have engaged in. The AMs were calibrated according to the manufacturers recommendations prior to each of the measurements.

### Estimating adherence and identifying a subset of highly adherent subjects

If a subject takes an AM off and it remains stationary, the AM will record a string of continuous zeroes. However, single zeroes or short strings of zeroes are not indicative of non-wear, because zeroes are a common occurrence when AMs are being worn. To estimate adherence, we utilized the criterion that a 20 min string of continuous zeroes represents an estimate of a non-wear occasion of an AM [12,15].

To simulate the effects of removing AMs on a regular basis, we wished to identify a "reference database" of PA in a group of subjects that wore them for prolonged periods of time, then remove data in a manner similar to what would happen if an AM was removed [15]. Since AMs are commonly removed by subjects during sleep and periodically during the day (e.g., showering, personal care), it is rare to find subjects that wear an AM 24 hr/day for more than a couple of consecutive days. Therefore, our criterion was to identify subjects that wore an AM for a minimum of 20 hrs/d for seven consecutive days. Of the 524 subjects, 18 women and 17 men fit the criterion (24 HR).

### Simulation of missing data (24 HR; Table 2)

**Table 2:** Description of simulations performed on a subset of highly adherent subjects (24 HR; n = 35).

Simulation	# of Simulations/subject	Description
A1	7	sleeping hours replaced with 0s
B1	7	sleeping hours deleted (waking hours only)
C1	7	sleeping hours replaced with a constant (23.1)
A2	133	A1 plus replacing missing waking hours with 0s
B2	133	B1 plus replacing missing waking hours with 0s
C2	133	C1 plus replacing missing waking hours with 0s
D	133	C1 plus imputing values for missing waking hours

**Figure 1:**
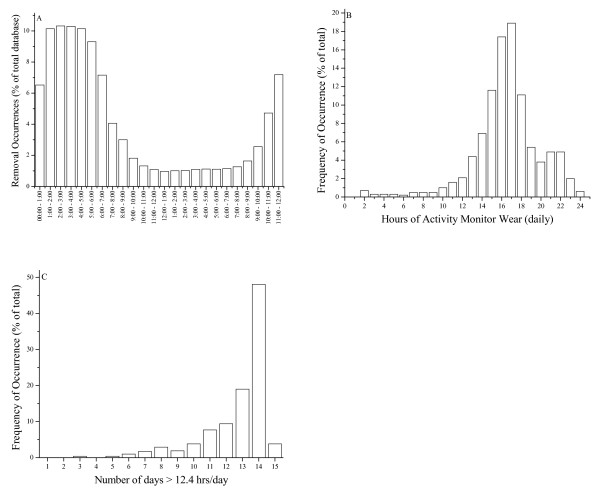
**Characteristics of estimated activity monitor wear adherence for a large-study of free-living adults (N = 523)**. Hours of Activity Monitor Wear = estimate of how many hours of a day an activity monitor was worn. Panel A demonstrates the time of day where activity monitor removals occurred for all of the daily records. Panel B is a histogram demonstrating the hours of activity monitor wear (per day) for all of the daily records. Panel C is a histogram demonstrating the number of days of data (assuming a "day" is more than 12.4 hrs/day) for all of the daily records.

There are two general instances when an AM is removed by a subject: 1) during sleep, and 2) intermittently during waking hours (Figure 1A). Therefore in our simulation study, we wished to differentiate between AM removal during sleeping and waking hours in the 24 HR dataset.

The first step in this simulation study was to estimate when subjects woke up and went to sleep using the data collected from the AM (not using subject self-report). These estimates were necessary because preliminary analyses indicated significant inconsistencies between self-reported waking and sleeping times, and obvious movement in the AM dataset. Also, subjects occasionally failed to report when they woke up or went to sleep. Since there are no sleep detection procedures for the waist-worn Actigraph AM, we developed a computer program using SAS([28](modified from Sadeh et al. [29]). The predicted waking and sleeping times from the computer program were compared to those reported by the subjects and by visually inspecting the daily data on a relative scale (positive and negative signs included) and an absolute scale (positive and negative signs ignored). The computer program predicted waking and sleeping times (relative and absolute differences) within -3.6 (30.8) minutes and +34.9 (60.3) minutes, respectively, when compared to those reported by the subjects in their daily activity logs. The computer program predicted waking and sleeping times within -6.0 (16.0) minutes and +34.6 (36.0) minutes, respectively, when compared to visual inspection of the data. The differences between the visual inspection of the data and self-report were -5.4 (32.3) minutes for waking times and +2.1 (41.0) minutes for sleeping times.

The second step was to simulate the effect of data loss due to AM removal during sleep. There are two general ways investigators treat the zeroes produced during sleep when AMs are removed: (1) include the zeroes in all estimates of PA [18,24,30], or (2) measure PA during waking hours only by removing the data observed during sleep [16,31,32]. Therefore, to simulate the effect of AM removal, we replaced the minute-by-minute data recorded during sleep from the 24 HR database with zeroes (Simulation A1), or deleted the sleeping hours from the database all together (Simulation B1).

The third step was to simulate AM removal during waking hours by putting zeroes in hourly blocks of time in the place of the raw data. These one hour blocks of time (10 per day for each of the seven days of data) were spread throughout the waking hours of the day, and randomly distributed between each of the seven days. Simulations were generated for single hourly blocks of time, and for multiple hours of time up to ten hours.

**Figure 2:**
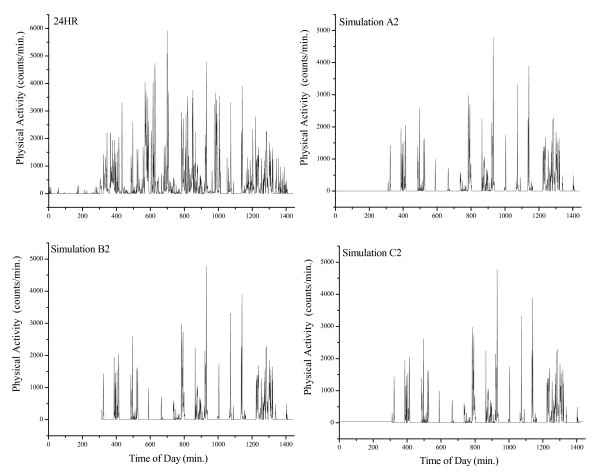
**Demonstration of activity monitor removal simulations during sleep and 10 waking hours from a single day**. 24 HR: raw data for 24 hours of a day (in minutes). Simulation A2: demonstration of removing an activity monitor for sleep and 10 waking hours (zeroes imputed in the place of the raw data). Simulation B2: demonstration of measuring only waking hours (raw data from sleep was deleted) and removing an activity monitor for 10 waking hours (zeroes imputed in the place of the raw data). Simulation C2: demonstration of removing an activity monitor for sleep (but imputing a constant value of 23.1 counts/min) and 10 waking hours (zeroes imputed in the place of the raw data).

In Simulation A2, we replaced the raw data observed during sleep and the hourly blocks of time during waking hours with zeroes. In Simulation B2, raw data recorded during sleep were deleted altogether (like Simulation B1) and zeroes replaced the missing hourly blocks of time during waking hours. Figure 2 demonstrates what a single day of minute by minute raw data from a random subject looks like (24 HR), and 10 hours of simulated AM removal during waking hours for the same subject and day.

Last, we compared the estimates of PA (counts/min) from the different simulations and total energy expenditure (MJ/day) measured by the doubly labeled water technique (see below) to the 24 HR database.

### Use of imputation to correct for missing data (24 HR; Table 2)

If missing data create biased estimates of PA, then substituting some reasonable value by imputation should improve the estimates of PA [15]. To estimate an imputation value for AM removal during sleep, we took the mean value for PA observed during sleep from the 24 HR database (23.1 counts/min), then imputed this value into the 24 HR database (Simulation C1). We also imputed this value for sleep and compared the sleep imputations to Simulations A2 and B2, where hourly blocks of time were removed from waking hours (Simulation C2).

To estimate the effect of correcting for AM removal during waking hours, we generated imputation estimates using a robust geostatistical technique, kriging [33]. Kriging, which has well established statistical properties, such as minimizing mean squared error under fairly general conditions, is used in spatial data for making predictions at locations for which data were not collected, based on the covariance structure of the observed locations. This prediction method is available in the SAS proc mixed software [28]. One must choose a covariance structure to use kriging. We selected an exponential decay model, so that the autocorrelation between neighboring one-hour AM averages decreased exponentially with increasing time separating the observations. This approach is analogous to the autoregressive (1) time series model for unequally spaced observations (missing data could occur at any waking time point). Predictions were generated using only data from that day of the subject's record. These imputations were used to substitute for the missing data, and were combined with imputation for sleeping hours (Simulation D). While many other models are available, in practice, for the small (one day) data sets actually used as support for the imputed data, different models would produce statistically indistinguishable results and, as described above, the exponential decay model is a reasonable choice.

### Resting Energy Expenditure (REE)

Resting energy expenditure (REE) was measured by respiratory gas analysis using a ventilated hood for 30 minutes in the early morning after a 12 hour overnight fast and 15 min of sitting quietly [34]. Energy expenditure was calculated by using the calculations of Weir [35].

### Total Energy Expenditure (TEE)

Another way to assess the effect of missing data on the prediction of PA is to examine its effect on the calculation of TEE for a two week period, where TEE was estimated using doubly labeled water (DLW) [36]. Subjects reported to the laboratory on the first day of the study between 6:30 and 9:00 a.m., at which time they received an oral dose of H_2_^18^O (0.08 g/kg body weight) and ^2^H_2_O (0.10 g/kg body weight). Urine samples were collected in the laboratory immediately before the dose and on four different mornings (second void) during the observation period. Two additional urine samples were collected away from the laboratory on days different from those collected in the lab. Thus, six urine samples were available for the analysis. Enrichments of ^2^H and ^18^O in urine samples were measured by isotope ratio mass spectrometry (Europa Scientific Hydra)[37]. TEE was calculated using the equation described by Weber et al. [38], which is based on the multipoint method described by Schoeller [39,40], dilution space adjustment described by Racette et al. [41], and the energy equivalent of carbon dioxide (5.6535)[42] based on an assumed respiratory quotient of 0.86.

To estimate TEE from PA data, linear regression equations were generated from the 24 HR database (proc mixed procedure from SAS [28]) using body weight, lean body mass, REE, PA, and different interactions between these variables. A number of different models were explored to identify the best choice, with the lowest value of AIC (Akaike's Information Criterion)[43] used as the criterion. The model with the lowest AIC had the independent variables, lean body mass, and the interaction between REE and PA (we included the REE and PA main effects when working with this model). We conducted a variance decomposition on the total variance of TEE from this model using estimated mean squares from a Type I ANOVA Table, calculated using the proc mixed procedure from SAS [28], and methods described in Searle et al. [44].

To estimate the effect of AM removal and/or the imputation procedures, TEE was estimated by substituting the PA from 24 HR with the estimates from the different simulations (A1, A2, B1, B2, C1, C2, and D).

### Statistics

Differences in the mean values for PA and TEE between the 24 HR reference dataset and the different simulations were compared using paired *t*-tests (paired by subject) and/or coefficients of variation (CV; standard deviation of the difference between 24 HR and a simulation divided by the mean, multiplied by 100). Preliminary analyses indicated the some of the data were not normally distributed [45], so analyses were performed on both raw and log-transformed data (log-transforming the daily means, which normalizes their distribution).

## Results

### Patterns of AM adherence in a large dataset of adults (n = 524)

Adherence was estimated to be 15.8 ± 3.4 hrs/day, with a range of 1.3 to 24.0 hrs/day (Fig 1B). Adherence was minimally influenced by the selection of the 20 min cut-off (15.5 ± 3.3 hrs/day for 15 min to 16.3 ± 3.6 hrs/day for 30 min). The estimated adherence by day of week was 14.9 (0.1), 16.1 (0.1), 16.1 (0.1), 16.1(0.1), 16.2 (0.1), 16.3 (0.1), and 15.0 (0.1) hrs/day (Sunday to Saturday). Sunday and Saturday adherences were significantly lower than each of the weekdays (p < 0.0001), but Sunday and Saturday were not different from each other, as were each of the weekdays also not different from each other. Lastly, gender differences in adherence were virtually non-existent. The data from one of the subjects were lost, due to an AM malfunction.

There is no consensus in the literature regarding many hours a day a subject must wear an AM to represent a "day", so we define a day as being 12.4 hrs. This definition of a day was calculated by removing days that were lower than 1 SD below the mean (based on a mean adherence of 15.8 ± 3.4 hrs/day). After omitting days where adherence was less than 12.4 hrs/day, daily adherence was 11.7 ± 2.0 days (out of 13 to 15 possible)(Fig 1C). Figure 1A indicates that although most AM removals likely occurred during sleep, approximately 30% of the removals occurred between 6:00 am and 10:00 pm.

### Simulating the effect of AM removal on predictions of PA and TEE (Table 3; 24 HR database)

**Table 3:** Effect of activity monitor removals during sleep on the prediction of physical activity and total energy expenditure.

	Physical Activity			
	counts·min^-1^·day^-1^		log (counts·min^-1^·day^-1^)	
Simulation	Mean (SD)	CV (%)	Mean (SD)	CV (%)
24 HR	228.4 (97.2)	-	5.35 (0.40)	-
A1	220.5 (97.6)*	3.1	5.30 (0.45)*	0.7
B1	332.9 (151.3)*	26.0	5.71 (0.43)*	4.8
C1	228.2 (98.8)	1.6	5.35 (0.42)	0.3

	Total Energy Expenditure			
	MJ·day^-1^		log (MJ·day^-1^)	
Simulation	Mean (SD)	CV (%)	Mean (SD)	CV (%)

24 HR	11.2 (3.3)	-	2.38 (0.29)	-
A1	11.2 (2.8)	9.0	2.37 (0.24)	4.1
B1	11.8 (4.2)	9.9	2.41 (0.26)	4.0
C1	11.2 (2.9)	9.0	2.38 (0.24)	4.0

*p < 0.05 vs. 24 HR

24 HR: physical activity data from subset of highly adherent subjects (n = 35). Simulation A1: simulation of activity monitor removal during sleep by imputing zeroes. Simulation B1: simulation of compensating for activity monitor removal during sleep by measuring waking hours only (sleeping physical activity deleted). Simulation C1: simulation of compensating for activity monitor removal during sleep by imputing a constant (23.1 counts/min) CV(%)= coefficient of variation = ((standard deviation of 24 HR – Simulation A1/B1/C1)/(mean of 24 HR and Simulation A1/B1/C1)) × 100.

When compared to 24 HR, imputing zeroes for sleep (Simulation A1) and deleting sleeping hours (Simulation B1) resulted in significant under-and over predictions of PA (p < 0.05), respectively, while imputing a constant value for sleeping hours (Simulation C1) reduced the bias. The under- and over-predictions of PA did not have a substantial effect on the prediction of TEE. Log-transformation of the data reduced the CV in all simulations.

### Simulating the effect of AM removal during sleeping and waking hours (Figure 3; 24 HR database)

**Figure 3:**
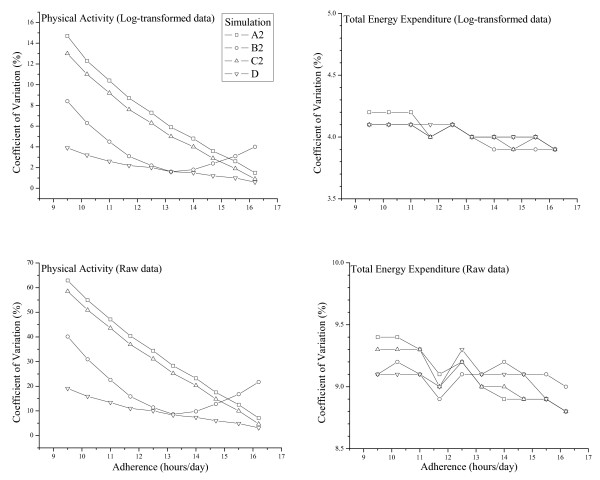
**Comparing the physical activity and total energy expenditure estimates of highly adherent subjects (24 HR; n = 35) to activity monitor removal simulations**. Simulation A2: simulation of activity monitor removal during sleep by imputing zeroes and during 1 and 10 waking hours (zeroes imputed in the place of the raw data). Simulation B2: simulation of compensating for activity monitor removal during sleep by measuring waking hours only (sleeping physical activity deleted), and during 1 and 10 waking hours (zeroes imputed in the place of the raw data). Simulation C2: simulation of compensating for activity monitor removal during sleep by imputing a constant (23.1 counts/min), and during 1 and 10 waking hours (zeroes imputed in the place of the raw data). Simulation D: simulation of compensating for activity monitor removal during sleep by imputing a constant (23.1 counts/min), and during 1 and 10 waking hours (imputing estimates in the place of the raw data). CV(%

Simulation of an AM removal (by imputing zeroes) for a single waking hour in Simulation A2 (zeroes imputed for sleep) resulted in a CV of approximately 7%, with each additional hour of missing data resulting in an increase of 5 – 10% per hour. A single hour of AM removal during waking hours resulted in a CV of approximately 21% for Simulation B2 (sleeping hours deleted). The additional hourly AM removals in B2 resulted in a curvilinear increase in the CV, where two to six hours of removals actually produced a lower CV than the CV produced with only a single hour deleted. This occurred because the different ways missing sleeping and waking hours were handled tended to offset each other when 2 – 6 hrs of waking data were removed.

The consistently lowest CV's throughout the entire range of missing data was observed for Simulation D (imputation of a constant for sleep and imputation of missing data during waking hours). We found the autoregressive parameter significant in 68.5% of the simulations (Wald test) when only one hour was imputed, but this value decreased to 39.2% when 10 hours was imputed. This means that our imputation procedure was effective in a majority of the cases when missing data were observed (although the effectiveness decreases as missing data increase).

As with the results from Table 3, log-transforming the data reduced the CV in all simulations.

### Variance decomposition of the model predicting TEE (24 HR database)

The largest component of explained variance was lean body mass (71.5%). The contribution of the sum of other effects was small (7.1%), composed of PA (0%), REE (2.0%) and the interaction between PA and REE (5.1%). Unexplained variation (by the model) was 21.3% of the total.

## Discussion

Although the adherence to a two week study of PA using AMs in a large study of free-living adults was rather strong, these analyses confirm that missing data can have a significant impact on the estimates of PA [15]. These data simulations demonstrate how critical it is for investigators to develop procedures that encourage subjects to wear AMs as much as possible. Fortunately, it appears as though the effects that missing data have on the estimates of PA can be significantly reduced by standard imputation procedures.

To give a simple example of the importance of AM data analysis procedures, take the example of a subject (#1) who accumulates 300,000 counts over the course of 17 hrs and takes the AM off for sleep during the remainder of the 24 hrs (zeroes remain in the dataset). Another subject (#2) accumulates 10% less total PA during the same 17 hr day, but total PA is measured during waking hours only (zeroes are deleted). The end result of the comparison is that although subject#1 accumulated 10% greater total PA, the counts/min average for subject#2 (265 counts/min) would be 27% higher than subject#1 (208 counts/min). If a constant for sleep is imputed for both subjects, both total (counts/day) and average (counts/min) PA are 10% greater for subject#1 vs. subject#2.

We suggest the following approach to minimize the effects of AM removals (Simulation D). First, identify the sleeping hours and substitute the zeroes with a constant. Then implement the kriging procedure to compensate for missing data observed during waking hours. Fortunately, it is not necessary to have our procedures in hand to make the imputations. For example, the identification of sleeping and waking times does not hinge on the use of our procedure; it is feasible to use a combination of subject self-report and visual identification from the data. Daily PA estimates when using our computer-generated wake/sleep estimates and visual identification resulted in small differences (CV of 0.7%). The advantage of the computer program is that the procedure is much faster. Also, there is no way of knowing whether the imputation value for sleep we use (23.1 counts/min) is the best for all ages and AM brands, but it appears to be sufficient for this population. The software and codes necessary to carry out the kriging procedure are widely available (in many statistical packages, and/or the principal investigator) and does not require a profound understanding of imputational statistics.

It is worth noting that problematic estimates resulting from missing data did not have large effects on the prediction of TEE. An explanation for this comes from the variance decomposition, where PA explained 0% variance by itself, and only 5.1% of the variance in its interaction with REE. While PA estimates do aid in the prediction of TEE from DLW, the large variance of the estimates of PA make this variable less useful to the model. The fact that PA data from AMs do not predict TEE well has also been reported by others [18,46].

A short comparison of the imputation methods used by Catellier et al. [15] with the one we used may be helpful. They are both variants of maximum likelihood methods, based on using a mean and known covariance structure, **Σ**. In our method, we assume **Σ **(which is separately estimated for one subject for one day) is not σ^2^**I**, that is, the hourly AM counts are not independent, but are correlated and the form of the correlation is known. Their imputation method simultaneously uses all the data, i.e. their **Σ **is based on all observations of all subjects over all days, so is much larger, and observations may or may not be assumed independent. They did not specify the form of **Σ **in their paper, and it may be complicated. We found that using data from other days of the same subject did not improve estimates [47], but this may not be true for other data sets. The data used by Catellier et al. [15] came from school age children, who are likely to have a more regimented day (thus more predictable) imposed on them by their school schedule, than our free-living adults. Both sets of predictions are based on conditional expected values, ours came entirely from the specified correlation structure of the data (two parameters) and the mean (one parameter), theirs may include several parameters characterizing the mean vector and the covariance structure.

Like any imputation method, good results can only be obtained if the pattern of the data captured by the model (in our case, the modeled covariance structure) reasonably approximates the true pattern generating the data. If there is little pattern to the data, then the best estimate is the mean. In fact, we found most estimates of the autocorrelation parameter significant, suggesting that knowledge of **Σ **was useful for obtaining estimates of missing values (i.e. better than simply substituting in the mean). Catellier et al. [15] went one step further, rather than simply replacing the missing data with an expected value (what we did), which tends to underestimate the true variance (of the complete data set, had there been no missing values), they used a sample from a generated distribution that they believed matched the true distribution of the missing data, so that variances are not downward biased. This was not necessary for us to be able to illustrate the points we wanted to make.

## Conclusion

Despite the potential problems of AM removals to the prediction of PA, it appears as though relatively simple imputation procedures can be implemented to reduce poor estimates. It should be noted that these procedures are effective in improving otherwise poor estimates of PA, but there are diminishing returns as the amount of missing data increases (Figure 3). It must also be noted that these imputations (particularly during waking hours) are only effective for correcting missing hourly blocks of time. These imputations cannot be carried out for minute by minute data, which is commonly used by investigators to estimate time spent in moderate PA. This restriction is because during short periods of time in waking hours, it is not clear if the observed zeroes in the data are due to inactivity or AM removal. Future investigations must refine these procedures to improve the estimates of shorter periods of missing AM time.

## Abbreviations

AM: Activity Monitor; PA: Physical Activity; TEE: Total Energy Expenditure; REE: Resting Energy Expenditure; 24 HR: Reference database of highly adherent subjects.

## Competing interests

The authors declare that they have no competing interests.

## Authors' contributions

DRP was responsible for data collection, statistical analysis and manuscript preparation. MK was responsible for statistical analysis and manuscript preparation. KSS and KES were responsible for data collection and manuscript preparation. AJM, DJB, and WVR were responsible for the study design and manuscript preparation. All authors have read and approved the final manuscript.

## Pre-publication history

The pre-publication history for this paper can be accessed here:



## References

[B1] Brooks GA, Butte NF, Rand WM, Flatt JP, Caballero B (2004). Chronicle of the Institute of Medicine physical activity recommendation: how a physical activity recommendation came to be among dietary recommendations. Am J Clin Nutr.

[B2] U.S. Department of Health and Human Services (1996). Physical Activity and Health: A Report of the Surgeon General.

[B3] Warburton DE, Nicol CW, Bredin SS (2006). Health benefits of physical activity: the evidence. CMAJ.

[B4] Pate RR, Pratt M, Blair SN, Haskell WL, Macera CA, Bouchard C, Buchner D, Ettinger W, Heath GW, King AC (1995). Physical activity and public health. A recommendation from the Centers for Disease Control and Prevention and the American College of Sports Medicine. JAMA.

[B5] National Academy of Sciences (2002). Dietary reference intakes for energy, carbohydrates, fiber, fat, protein, and amino acids (macronutrients). National Academy of Sciences, Institute of Medicine.

[B6] Troiano RP, Macera CA, Ballard-Barbash R (2001). Be physically active each day. How can we know?. J Nutr.

[B7] Mahabir S, Baer DJ, Giffen C, Clevidence BA, Campbell WS, Taylor PR, Hartman TJ (2006). Comparison of energy expenditure estimates from 4 physical activity questionnaires with doubly labeled water estimates in postmenopausal women. Am J Clin Nutr.

[B8] Kesaniemi YK, Danforth E, Jensen MD, Kopelman PG, Lefebvre P, Reeder BA (2001). Dose-response issues concerning physical activity and health: an evidence-based symposium. Med Sci Sports Exerc.

[B9] Paul DR, Rhodes DG, Kramer M, Baer DJ, Rumpler WV (2005). Validation of a food frequency questionnaire by direct measurement of habitual ad libitum food intake. Am J Epidemiol.

[B10] Ward DS, Evenson KR, Vaughn A, Rodgers AB, Troiano RP (2005). Accelerometer use in physical activity: best practices and research recommendations. Med Sci Sports Exerc.

[B11] Troiano RP (2005). A timely meeting: objective measurement of physical activity. Med Sci Sports Exerc.

[B12] Treuth MS, Sherwood NE, Baranowski T, Butte NF, Jacobs DR, McClanahan B, Gao S, Rochon J, Zhou A, Robinson TN (2004). Physical activity self-report and accelerometry measures from the Girls health Enrichment Multi-site Studies. Prev Med.

[B13] Stevens J, Suchindran C, Ring K, Baggett CD, Jobe JB, Story M, Thompson J, Going SB, Caballero B (2004). Physical activity as a predictor of body composition in American Indian children. Obes Res.

[B14] Yoshioka M, Ayabe M, Yahiro T, Higuchi H, Higaki Y, St-Amand J, Miyazaki H, Yoshitake Y, Shindo M, Tanaka H (2005). Long-period accelerometer monitoring shows the role of physical activity in overweight and obesity. Int J Obes Relat Metab Disord.

[B15] Catellier DJ, Hannan PJ, Murray DM, Addy CL, Conway TL, Yang S, Rice JC (2005). Imputation of missing data when measuring physical activity by accelerometry. Med Sci Sports Exerc.

[B16] Goris AH, Meijer EP, Kester A, Westerterp KR (2001). Use of a triaxial accelerometer to validate reported food intakes. Am J Clin Nutr.

[B17] Matthews CE, Freedson PS (1995). Field trial of a three-dimensional activity monitor: comparison with self report. Med Sci Sports Exerc.

[B18] Masse LC, Fulton JE, Watson KL, Mahar MT, Meyers MC, Wong WW (2004). Influence of body composition on physical activity validation studies using doubly labeled water. J Appl Physiol.

[B19] Lof M, Hannestad U, Forsum E (2003). Comparison of commonly used procedures, including the doubly-labelled water technique, in the estimation of total energy expenditure of women with special reference to the significance of body fatness. Br J Nutr.

[B20] Paschali AA, Goodrick GK, Kalantzi-Azizi A, Papadatou D, Balasubramanyam A (2005). Accelerometer feedback to promote physical activity in adults with type 2 diabetes: a pilot study. Percept Mot Skills.

[B21] Atkinson JL, Sallis JF, Saelens BE, Cain KL, Black JB (2005). The association of neighborhood design and recreational environments with physical activity. Am J Health Promot.

[B22] Vogels N, Egger G, Plasqui G, Westerterp KR (2004). Estimating changes in daily physical activity levels over time: implication for health interventions from a novel approach. Int J Sports Med.

[B23] Meijer EP, Westerterp KR, Verstappen FT (2000). Effect of exercise training on physical activity and substrate utilization in the elderly. Int J Sports Med.

[B24] Ainsworth BE, Bassett DR, Strath SJ, Swartz AM, O'Brien WL, Thompson RW, Jones DA, Macera CA, Kimsey CD (2000). Comparison of three methods for measuring the time spent in physical activity. Med Sci Sports Exerc.

[B25] Schoeller DA, Tylavsky FA, Baer DJ, Chumlea WC, Earthman CP, Fuerst T, Harris TB, Heymsfield SB, Horlick M, Lohman TG (2005). QDR 4500A dual-energy X-ray absorptiometer underestimates fat mass in comparison with criterion methods in adults. Am J Clin Nutr.

[B26] Chen KY, Bassett DR (2005). The technology of accelerometry-based activity monitors: current and future. Med Sci Sports Exerc.

[B27] Bouchard C, Tremblay A, Leblanc C, Lortie G, Savard R, Theriault G (1983). A method to assess energy expenditure in children and adults. Am J Clin Nutr.

[B28] SAS Institute Inc

[B29] Sadeh A, Sharkey KM, Carskadon MA (1994). Activity-based sleep-wake identification: an empirical test of methodogical issues. Sleep.

[B30] Hoos MB, Kuipers H, Gerver WJ, Westerterp KR (2004). Physical activity pattern of children assessed by triaxial accelerometry. Eur J Clin Nutr.

[B31] Page A, Cooper AR, Stamatakis E, Foster LJ, Crowne EC, Sabin M, Shield JP (2005). Physical activity patterns in nonobese and obese children assessed using minute-by-minute accelerometry. Int J Obes Relat Metab Disord.

[B32] Matthews CE, Ainsworth BE, Thompson RW, Bassett DR (2002). Sources of variance in daily physical activity levels as measured by an accelerometer. Med Sci Sports Exerc.

[B33] Cressie NAC (1993). Statistics for spatial data, revised edition.

[B34] Howe J, Rumpler W, Seale J (1993). Energy expenditure by indirect calorimetry in premenopausal women: variation within one menstrual cycle. J Nutr Biochem.

[B35] Weir JB (1949). New methods for calculating metabolic rate with special reference to protein metabolism. J Physiol.

[B36] Speakman J (1997). Doubly labelled water: theory and practice.

[B37] Blanton CA, Moshfegh AJ, Baer DJ, Kretsch MJ (2006). The USDA Automated Multiple-Pass Method accurately estimates group total energy and nutrient intake. J Nutr.

[B38] Weber JL, Reid PM, Greaves KA, DeLany JP, Stanford VA, Going SB, Howell WH, Houtkooper LB (2001). Validity of self-reported energy intake in lean and obese young women, using two nutrient databases, compared with total energy expenditure assessed by doubly labeled water. Eur J Clin Nutr.

[B39] Schoeller DA (1983). Energy expenditure from doubly labeled water: some fundamental considerations in humans. Am J Clin Nutr.

[B40] Schoeller DA (1988). Measurement of energy expenditure in free-living humans by using doubly labeled water. J Nutr.

[B41] Racette SB, Schoeller DA, Luke AH, Shay K, Hnilicka J, Kushner RF (1994). Relative dilution spaces of 2H- and 18O-labeled water in humans. Am J Physiol.

[B42] Elia M, Livesey G (1992). Energy expenditure and fuel selection in biological systems: the theory and practice of calculations based on indirect calorimetry and tracer methods. World Rev Nutr Diet.

[B43] Burnham KP, Anderson DR (1998). Model selection and inference: A practical information-theoretic approach.

[B44] Searle SR, Casella G, McCulloch CE (1992). Variance Components.

[B45] White H (1980). A heteroskedastic-consistent covariance matrix estimator and a direct test of heteroskedasticity. Econometrica.

[B46] Leenders NY, Sherman WM, Nagaraja HN (2006). Energy expenditure estimated by accelerometry and doubly labeled water: do they agree?. Med Sci Sports Exerc.

[B47] Paul DR, Kramer M, Moshfegh AJ, Baer DJ, Rumpler WV (2005). Improving acceptability criteria for epidemiological physical activity monitor data. Federation of American Societies for Experimental Biology Conference.

